# Association of sleep quality before and after SARS-CoV-2 infection with clinical outcomes in hospitalized patients with COVID-19 in China

**DOI:** 10.17179/excli2021-3451

**Published:** 2021-04-14

**Authors:** Li Zhang, Tingting Li, Liangkai Chen, Feng Wu, Wenguang Xia, Min Huang, Zhenli Guo, Lin Song, Hongxiang Yin, Yangpu Zhang, Yongfei Yu, Sijie Cai, Zijian Lu, Shuang Rong, Wei Bao

**Affiliations:** 1 Department of Neurology, Hubei Provincial Hospital of Integrated Chinese & Western Medicine, Wuhan 430015, China; 2 Department of Nutrition and Food Hygiene, School of Public Health, Medical College, Wuhan University of Science and Technology, Wuhan 430065, China; 3 Department of Nutrition and Food Hygiene, Hubei Key Laboratory of Food Nutrition and Safety, Ministry of Education Key Lab of Environment and Health, School of Public Health, Tongji Medical College, Huazhong University of Science and Technology, Wuhan 430030, China; 4 Department of Epidemiology, College of Public Health, University of Iowa, Iowa City, IA 52242, USA

**Keywords:** Coronavirus disease 2019, SARS-CoV-2, sleep, epidemiology

## Abstract

Sleep is believed to benefit the host defense against pathogens. We aimed to investigate the association of sleep quality with clinical outcomes among hospitalized patients with COVID-19. We conducted a prospective cohort study in 205 adult hospitalized patients with diagnosed moderate COVID-19, with follow-up until hospital discharge or death. Pittsburgh Sleep Quality Index (PSQI) assessed sleep quality before and after infection. The primary outcome was the incidence of severe or critical pneumonia, and the secondary outcomes were duration of hospital stay and laboratory measurements during the follow up. Among the 205 included hospitalized patients, 185 (90.2 %) experienced poorer sleep quality after infection than before according to the PSQI score, and 25 (12.2 %) developed severe or critical pneumonia during follow-up. In Cox regression models, the adjusted hazard ratio of developing severe or critical pneumonia associated with each 1 score increment in the PSQI score before and after infection was 1.23 (95% CI: 1.09, 1.39) and 1.35 (95 % CI: 1.08, 1.67), respectively. Poorer sleep quality was also significantly associated with a prolonged hospital stay and more serious dysregulations in immune system indicated by several laboratory markers. Poorer sleep quality, either in the daily time or after infection with SARS-CoV-2, was associated with worse clinical outcomes. These findings highlight the importance of good sleep in confronting the emerging pandemic of COVID-19.

## Introduction

Coronavirus disease 2019 (COVID-19), caused by the severe acute respiratory syndrome coronavirus 2 (SARS-CoV-2) (Zhu et al., 2020[31]), was first recognized and reported in Wuhan, China in late December of 2019. The pandemic of coronavirus disease 2019 (COVID-19) is posing significant challenge to global public health. By March 26, 2021, over 124 million confirmed COVID-19 cases and 2.7 million deaths from COVID-19 have been reported worldwide (WHO, 2021[28]). According to data from China, although the case fatality rate among mild to moderate cases was low, the case fatality rate was as high as about 50 % in critical cases (Yang et al., 2020[30]; Wu and McGoogan, 2020[29]). It is imperative to identify risk factors that are related to clinical progression of COVID-19. 

Sleep may benefit the host defense against pathogens. Previous epidemiologic studies showed that sleep was beneficial to the host by maintaining a robust immune system and may have an association with healing and survival among those being infected (Patel et al., 2012[20]; Kuo and Williams, 2014[15]; Hahn et al., 2020[7]). 

As COVID-19 pandemic spreading worldwide, sleep quality and mental health of general population were severely affected due to home quarantine, isolation, and social distancing (Casagrande et al., 2020[4]; Salehinejad et al., 2020[25]). Sleep impairment was frequently reported among hospitalized patients with COVID-19 (Liguori et al., 2020[16]). In turn, poor sleep whether before or after infection may affect patients' disease progression and prognosis (Huang et al., 2020[8]; Akinci and Melek Basar, 2021[2]), although a study did not identify a significant contribution of sleep disorders to outcomes related to SARS-CoV-2 infection (Perger et al., 2021[21]). 

It is hypothesized that both habitual sleep quality and poor sleep quality after infection with SARS-CoV2 affect patient immunity and disease progression. Accordingly, the purpose of this study was to explore the association of sleep quality before and after infection with disease severity among hospitalized patients with COVID-19. 

## Methods

### Participants

**Figure 1:**
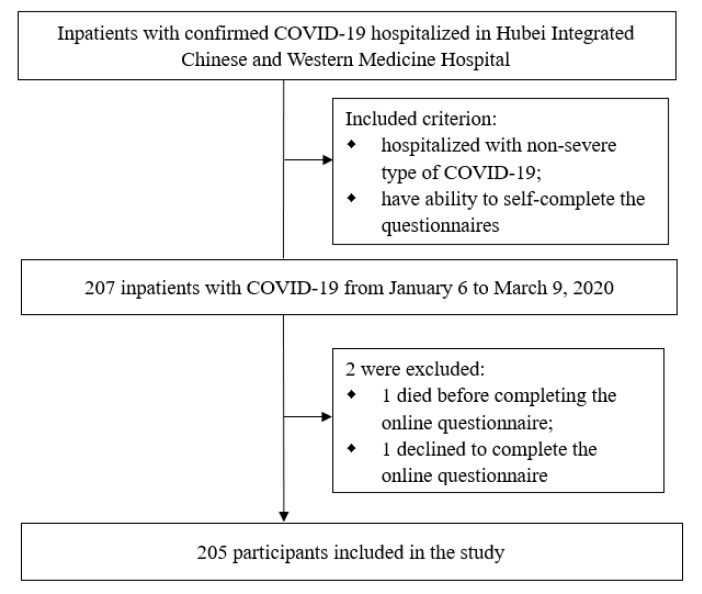
Flow diagram of the participants

We conducted a prospective cohort study among hospitalized adult patients with diagnosed moderate COVID-19 in Hubei Integrated Chinese and Western Medicine Hospital, one of the major designated hospitals for treating patients with COVID-19 in Wuhan, China. Case definitions of confirmed human infection with SARS-CoV-2 were in accordance with the interim guidance from the World Health Organization (WHO, 2021[27]). We enrolled 207 patients who were admitted to the hospital from January 6 to March 9, 2020 meeting the following criteria: laboratory confirmed SARS-CoV-2 infection, having fever and respiratory tract symptoms and pneumonia manifestations in imaging, clinically diagnosed as moderate cases of COVID-19 at admission, and having the ability to self-complete the questionnaires. These patients were followed up till the last patient discharged at March 30, 2020. Among them, 1 died before completing the questionnaire and 1 refused to participate. Finally, 205 patients completed the questionnaires and were included in this analysis (Figure 1(Fig. 1)). 

Neither patients nor the public were involved in the conception or conduct of the study. Written informed consent was obtained from each participant. The study was conducted according to the latest version of the Declaration of Helsinki ethical standards and approved by the Ethical Committee of the Hubei Provincial Hospital of Integrated Chinese (ethical approval code: 2020014). 

### Measures

Sleep quality was assessed using the adjusted items bases on Pittsburgh Sleep Quality Index (PSQI) (Buysse et al., 1989[3]), which is the most commonly used questionnaire to assess sleep health status in clinical and epidemiologic studies (Mollayeva et al., 2016[18]). PSQI is a self-administered scale with 18 items about sleep over the past 1 month, including seven domains of sleep (i.e., sleep quality, sleep onset latency, sleep duration, sleep efficiency, sleep disturbances, sleep medication, and daytime dysfunction). A sub score ranging from 0 to 3 is calculated for each of the seven domains, which can be summed up to yield a total score ranging from 0 to 21. A higher score represents worse sleep status. A total PSQI score of 0-5, 6-10, 11-15, and 16-21 was considered very good, fairly good, general, and poor sleep quality, respectively (Dobrosielski et al., 2016[5]). We used a slightly modified version, in which we deleted the time restriction “over the past 1 month” and added the terms “1 month before infection” and “after infection” to collect information about the patients' sleep status before and after infection with SARS-CoV-2. 

Anxiety and depression were assessed by the Hospital Anxiety and Depression Scale (HADS) (Zigmond and Snaith, 1983[32]). HADS consists of seven items measuring anxiety and seven items measuring depression, which are summed to form anxiety and depression subscale scores (HADS-A and HADS-D, respectively). All items are measured on a four-point scale (0-3) and refer to the past week.

### Clinical outcomes

In this study, the primary outcome was the incidence of severe or critical pneumonia, and the secondary outcomes were duration of hospital stay and laboratory measurements.

Electronic medical records were extracted and reviewed by a team of clinical doctors (L.Z., F.W., W.X., M.H., Z.G., L.S., H.Y., Y.Z., and Y.Y) who treated those patients with COVID-19 and double-checked by another team of researchers (T.L., S.C. and Z.L.). Information regarding clinical, laboratory, treatment, and outcomes were extracted from medical records using modified WHO/International Severe Acute Respiratory and Emerging Infection Consortium case record forms. We collected data on symptoms from onset to admission, underlying comorbidities (i.e., chronic pulmonary disease, hypertension, diabetes, cardiovascular disease, cerebrovascular disease, and chronic kidney disease), and laboratory findings. SARS-CoV-2 in nasopharyngeal swab specimens was detected by real-time RT-PCR (DAAN Gene, China). The illness severity of COVID-19 was defined according to the Chinese management guideline for COVID-19 (version 7) (National Health Commisson of the People's Republic of China, 2020[19]). Adults who met any of the following criteria were classified as severe cases: (a) respiratory rate ≥ 30 breaths/min; (b) oxygen saturation ≤ 93% at a rest state; (c) arterial partial pressure of oxygen (PaO_2_)/oxygen concentration (FiO_2_) ≤ 300 mmHg; (d) > 50 % lesions progression within 24 to 48 hours in lung imaging. Adults who meet any of the following criteria were classified as critical cases: (a) occurrence of respiratory failure requiring mechanical ventilation; (b) presence of shock; (c) other organ failure that requires monitoring and treatment in the ICU. Throat-swab specimens were obtained for SARS-CoV-2 RT-PCR re-examination every other day after clinical remission of symptoms. The discharge criteria were as follows: (a) normal temperature for 3 consecutive days; (b) symptom relief; (c) negative throat-swab specimens repeated twice with at least 1 day interval; and (d) significant improvement in exudative lesions in lung imaging. 

### Laboratory measurement

Laboratory markers were tracked from admission to discharge or death. Complete blood count, including white blood cell count (WBC), lymphocyte percentage and monocyte percentage, were assessed by double sheath flow laser counting method (Mindray-6800, China). Alanine aminotransferase (ALT) and lactate dehydrogenase (LDH) were detected by rate method and creatinine by enzyme method (Hitachi-7600, Japan). Procalcitonin (PCT) was measured by chemiluminescence method (Roche-411, Switzerland). Plasma D-dimer and serum amyloid A (SAA) by scattering turbidimetry (Mindray-XRM, China; Sunray-300, China). C-reactive protein (CRP) was assayed by immunoturbidimetry (Hitachi-7600, Japan) and interleukin-6 (IL-6), IL-8, and IL-10 by chemiluminescence method (Siemens IMMULITE^®^ 1000, Germany). In addition, lymphocyte subpopulations, including total T cells, total B cells, CD4+ T cells, CD8+ T cells were determined by flow cytometry (BriCyteE6, Mindray, China). All tests were performed according to the corresponding manufacturer's protocol.

### Procedure

To prevent the spread of COVID-19 and comply with the ethical protocol of the ethical board, data on demographic and lifestyle characteristics, sleep quality, and mental health from the patients were collected via self-administered electronic questionnaires. The assessment forms were completed within the second week of hospitalization. If the patients indicated a need for assistance or clarification to complete the questionnaires, remote assistance was provided by the physicians and researchers via telephone calls. Data quality and completeness of the questionnaire were checked daily. Clinical and laboratory testing data among the patients were also extracted from medical records.

### Statistical analysis

Continuous variables were expressed as mean (standard division) for variables with normal distribution or median (interquartile range [IQR]) for variables with skewed distribution, and they were compared using ANOVA test (normal distribution) or Kruskal Wallis test (skewed distribution). Categorical variables were summarized as n (%) and compared using Chi-square test or Fisher exact test when needed. The association of sleep quality with the risk of developing severe or critical pneumonia was estimated using Cox proportional hazards regression models with the following covariates: age, sex, education, smoking, alcohol intake, physical activity, BMI, hypertension, diabetes, CVD, anxiety, and depression. Follow up time was calculated as the difference between the admission date and the date of confirming severe case or the discharge date. Smoothing splines were generated by generalized additive models to present the association of PSQI score with duration of hospital stay after adjustment for age, sex, education, smoking, alcohol intake, physical activity, BMI, hypertension, diabetes, CVD, anxiety, and depression. All analyses were performed using R software (The R Foundation, http://www.r-project.org, version 3.6.1) and EmpowerStats (http://www.empowerstats.net/en/, X&Y Solutions, Inc., Boston, MA, USA). A two-tailed *p *value below 0.05 was considered statistically significant.

## Results

**Figure 2:**
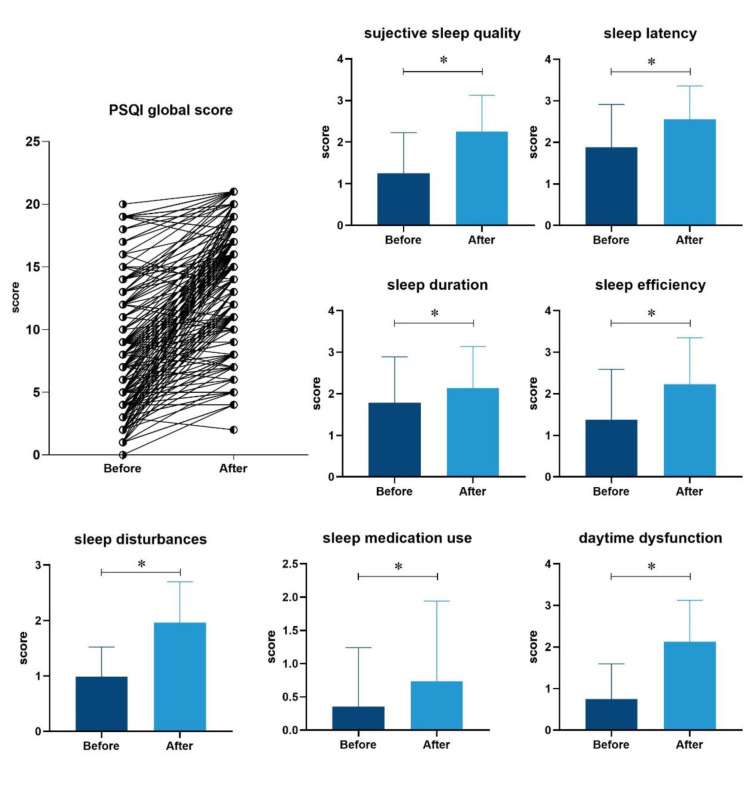
Comparison of PSQI score and its seven domains before and after SARS-CoV-2 infection. *: *p*-value < 0.001. The higher PSQI score represents the worse sleep quality.

Among the 205 hospitalized patients (mean age 58 years; 106 [51.7 %] were male) with moderate COVID-19 at admission, 25 (12.2 %) developed severe or critical pneumonia during the follow-up. Eventually, 202 survived and were discharged, and 3 died during hospitalization. The median of PSQI score among the included patients was 8 (IQR, 4-12) before infection and 15 (IQR, 11-17) after infection. According to the PSQI scores, 185 (90.2 %) hospitalized patients experienced poorer sleep quality than before infection. In general, there was a deterioration in each domain of sleep health status (i.e., sleep quality, sleep onset latency, sleep duration, sleep efficiency, sleep disturbances, sleep medication, and daytime dysfunction) after the patients were infected with SARS-CoV-2, as compared with their habitual sleep status before infection (Figure 2(Fig. 2)). Patients who had poorer habitual sleep quality before infection were older, more likely to have lower education level, have underlying hypertension, diabetes and cardiovascular disease (Table 1(Tab. 1)). 

**Figure 3:**
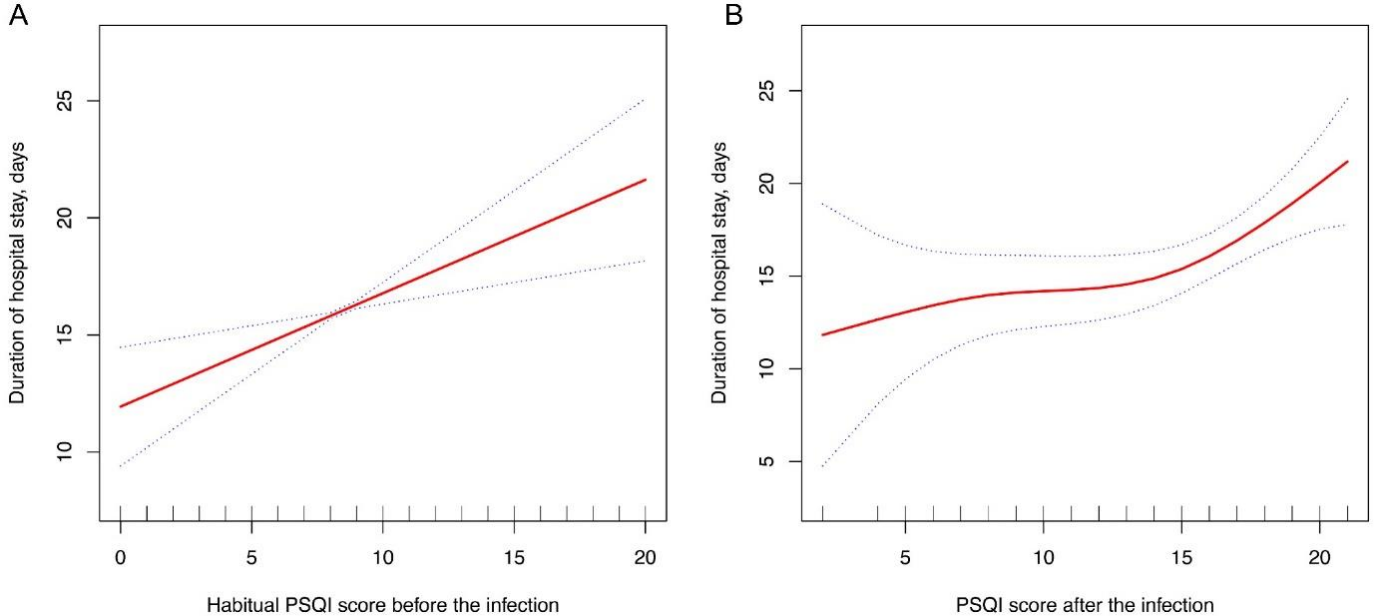
Association of PSQI score before and after infection with duration of hospital stay. Smoothing splines were generated by generalized additive models and adjusted for age, sex, education, smoking, alcohol intake, physical activity BMI, hypertension, diabetes, CVD, anxiety, and depression. The red line indicates the estimated duration of hospital stay, and the blue dot line indicates 95 % confidence intervals.

We observed a significant association of both sleep quality before and after infection with the risk of developing severe or critical pneumonia. In the multivariable Cox regression models, each 1 score increment of PSQI score before infection was associated with 23 % higher risk of developing severe or critical pneumonia (hazard ratio [HR], 1.23; 95 % CI: 1.09, 1.39) (Table 2(Tab. 2)). Similar findings were observed for the associations of PSQI score after infection with the risk of developing severe or critical pneumonia (HR, 1.35; 95 % CI: 1.08,1.67). In addition, both PSQI scores before and after infection were positively associated with the duration of hospital stay (Figure 3(Fig. 3)).

**Figure 4:**
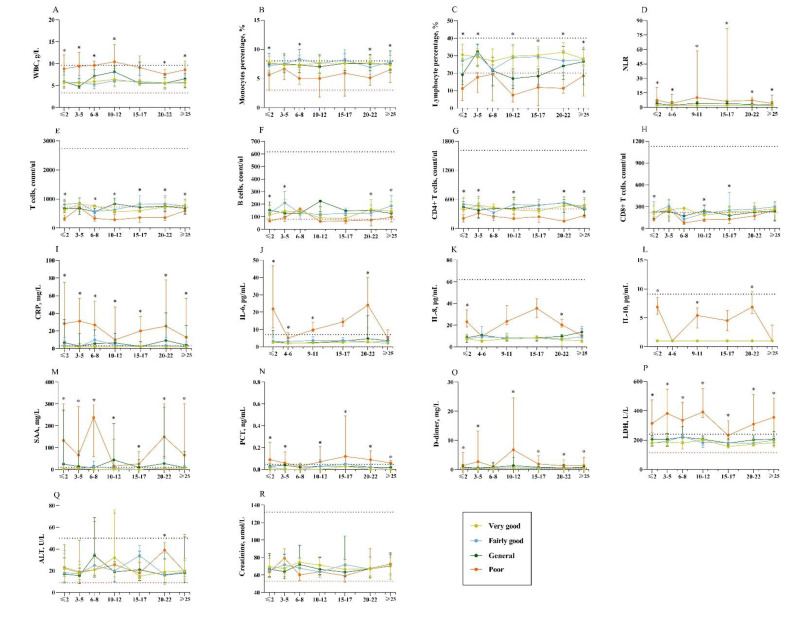
Temporal changes in laboratory markers from illness onset in hospitalized patients with COVID-19. Laboratory markers included white blood cell (WBC) (A), monocyte percentage (B), lymphocyte percentage (C), neutrophil-to-lymphocyte ratio (NLR) (D), T cells (E), B cells (F), CD4+ T cells (G), CD8+ T cells (H), C-reactive protein (CRP) (I), interleukin-6 (IL-6) (J), IL-8 (K), IL-10 (L), serum amyloid A (SAA) (M), procalcitonin (PCT) (N), D-dimer (O), lactate dehydrogenase (LDH) (P), alanine aminotransferase (ALT) (Q), and creatinine (R). The red dot line indicates the lower limit of the normal range and blue dot line indicates the upper limit of the normal range.

For laboratory markers, patients with poor sleep quality had a higher level of WBC, neutrophil-to-lymphocyte ratio (NLR), CRP, PCT, SAA, IL-6, IL-8, IL-10, LDH, D-dimer, and decreased lymphocyte count including T cells and B cells at baseline (Figure 4(Fig. 4)). During the whole course of disease, lymphocytes especially total T cells, CD4+ T cells, and CD8+ T cells were below the lower limit of the normal range (Figure 4(Fig. 4)). The levels of inflammation-related indicators (CRP, SAA, PCT, IL-6, IL-8, and IL-10), LDH, D-dimer and ALT were significantly elevated among patients with poor sleep quality compared with the other three groups (Figure 4(Fig. 4)).

## Discussion

In the present study, we examine the association of sleep quality with clinical outcomes and laboratory markers among 205 adult hospitalized patients with COVID-19. Our findings showed that poor sleep quality, both before and after infection, was independently associated with a high risk of developing severe or critical pneumonia. We also observed associations of poorer sleep quality before and after infection with a prolonged hospital stay. In addition, patients with poor sleep quality showed more serious dysregulation in immune system reflected by several laboratory markers.

Findings of this study were generally consistent with findings of recent studies or other previous infective diseases. Decreased sleep status and reduced sleep time in the last 7 days before the diagnosis of COVID-19 significantly increased risk of severe infection (Huang et al., 2020[8]). Patients with poor sleep quality during hospitalization were hospitalized for a significantly longer period than were those with good sleep quality, had higher depression rate (Akinci and Melek Basar, 2021[2]), and subjectively assessed themselves as being more severely sick (Jiang et al., 2021[14]). In a nationally representative sample of adults, self-reported short sleep duration and sleep disorders were associated with higher risks of a cold or infection or both in the past 30 days (Prather and Leung, 2016[23]). A prospective cohort study found that compared with non-shift workers, shift workers had a higher incidence rate of influenza-like illness/acute respiratory infection that was mainly mediated by poorer sleep quality (Loef et al., 2020[17]). A prospective study from Nurses' Health Study II cohort showed that self-reported short habitual sleep (<5 h per night) predicts pneumonia risk (Patel et al., 2012[20]), and short sleep duration was associated with increased susceptibility to the common cold (<6 h sleep per night) (Prather et al., 2015[22]). In addition, an animal study of bacterial infection challenge showed that mice that were allowed to sleep before systemic bacterial infection had increased survival upon infection (Hahn et al., 2020[7]), which supports potential effects of sleep on maintaining and/or enhancing immune function.

In addition, patients with poor sleep quality showed more serious dysregulation in immune system reflected by several laboratory markers. A retrospective study among 452 patients with COVID-19 suggested that COVID-19 might damage lymphocytes, especially T lymphocytes. The number of T cells significantly decreased, and more hampered in severe cases (Qin et al., 2020[24]). Our study extended those findings and showed that poor sleep quality may have further adverse effects on immune system among patients with COVID-19. Lymphocytes, especially total T cells, CD4+ T cells, and CD8+ T cells were decreased remarkably in patients with poor habitual sleep quality. Lymphocytes are necessary for the control of viral infection and correlated with disease progression (Huang et al., 2020[9]).

The observed association between poorer sleep quality and worse clinical outcomes among hospitalized patients with COVID-19 are biologically plausible. When an acute threat is perceived at the organismic or cellular level (such as viral exposure or cellular stress), increases in sleep duration is thought to occur to further augment host defenses (Irwin, 2019[11]). Among individuals with habitual sleep disturbance, increased inflammation may be caused at the expense of antiviral immune responses (Irwin, 2019[11]). A meta-analysis of more than 50,000 adults demonstrated that sleep disturbance is associated with higher levels of CRP and IL-6 (Irwin et al., 2016[13]). The sympathetic nervous system (SNS) and the hypothalamus-pituitary-adrenal (HPA) axis are the main effector systems that link sleep and immunity. Persistent activation of the HPA axis (Abell et al., 2016[1]) and a marked decrease in SNS activity (Irwin et al., 1999[10]) caused by sleep disturbance, but not acute sleep deprivation, have been revealed to shift the transcriptional profile toward increased inflammatory activity and decreased antiviral responses (Webster et al., 2001[26]; Irwin and Cole, 2011[12]). Further investigation is needed to determine the underlying mechanisms for the association between sleep quality and clinical outcomes among patients with COVID-19.

This study has important clinical and public health implications. The pandemic of COVID-19 is causing serious challenge to health care system, families, and society. Most of the cases with COVID-19 are mild or moderate, but some of them may progress to severe or critical disease (Gandhi et al., 2020[6]). Identifying risk factors that are associated with the progression of COVID-19 could help inform life-saving clinical decision-making or preventive measures. Given the observed association between sleep quality and clinical outcomes after COVID-19, getting good sleep may be one of the important resilience-related factors for the public and the patients to protect against adverse outcomes of COVID-19 pandemic.

This study has several strengths. The prospective cohort study design allows assessing temporal relationship of sleep quality with clinical outcomes and laboratory markers among adult hospitalized patients with moderate COVID-19. Moreover, sleep status before and after infection were both assessed to elaborate the associations of habitual and stress-related sleep with progression of COVID-19. In addition, we collected a variety of demographic, socioeconomic, lifestyle factors and therefore were able to control for potential confounding from these factors. There are some limitations. First, sleep quality was assessed by self-reports which may be subject to recall bias and misreporting. However, the PSQI that we used for assessing sleep status in this study has been previously validated and commonly used in clinical and research settings (Mollayeva et al., 2016[18]), and physicians and researchers in this study provided remote assisted guidance to ensure quality. Second, this study was based on a major hospital in Wuhan, China. Further investigation is needed to replicate our findings in other settings. Third, the observational nature of the present study might limit the capacity of causal inference.

## Conclusions

In summary, this study showed that among patients with COVID-19, poorer sleep quality, either before or after infection with SARS-CoV-2, was associated with worse clinical outcomes. Our study proposed an important message to individuals and society that keeping good sleep routine might contribute to confront this emerging pandemic and improve clinical outcomes. Additionally, monitoring sleep quality during hospitalization among COVID-19 patients may provide important information about the prognosis of COVID-19. Further investigation is needed to replicate our findings and determine the underlying mechanisms.

## Notes

Li Zhang, Tingting Li, Liangkai Chen and Feng Wu contributed equally as first author.

Shuang Rong and Wei Bao (Department of Epidemiology, College of Public Health, University of Iowa, 145 N Riverside Dr, Iowa City, IA 52242, USA; E-mail: wei-bao@uiowa.edu) contributed equally as corresponding author. 

## Acknowledgements

The authors thank Hui Lv, Yurong Liu, Liubo Ye, Zhaoxia Wang, Meiling Zheng, and Liang Tong for their contribution on assistance in data disposal. We thank all the patients enrolled in our study, mourn all the lives lost during this pandemic, and wish to pay tribute to all those currently fighting against COVID-19.

## Contributors

SR, LZ, and WB designed research. LZ, FW, WX, MH, ZG, LS, HY, YZ, and YY contributed to the acquisition, analysis, or interpretation of the data. All authors contributed to critically revise the manuscript for important intellectual content. SR has primary responsibility for final content and is the study guarantor. All authors read and approved the final manuscript. The corresponding authors attest that all listed authors meet authorship criteria and that no others meeting the criteria have been omitted.

## Funding

No funding was received for the present study.

## Competing interests

The authors declare no conflict of interest.

## Ethical approval

This study was approved by the Medical Ethics Committee of Hubei Integrated Chinese and Western Medicine Hospital and Wuhan University of Science and Technology.

## Figures and Tables

**Table 1:**
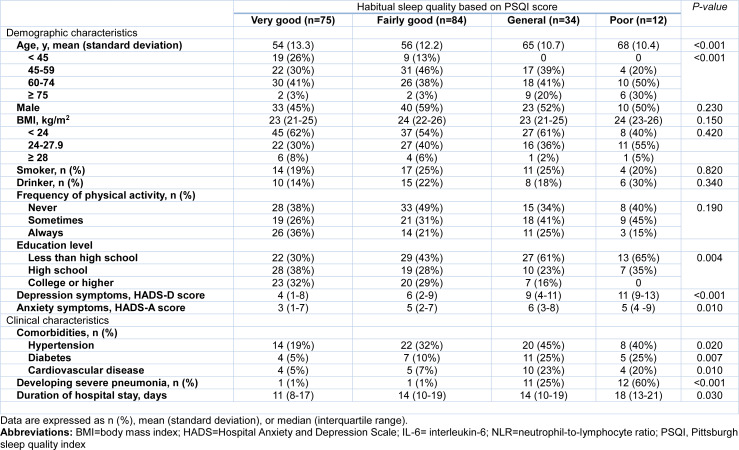
Demographic and clinical characteristics of 205 hospitalized patients with moderate COVID-19

**Table 2:**
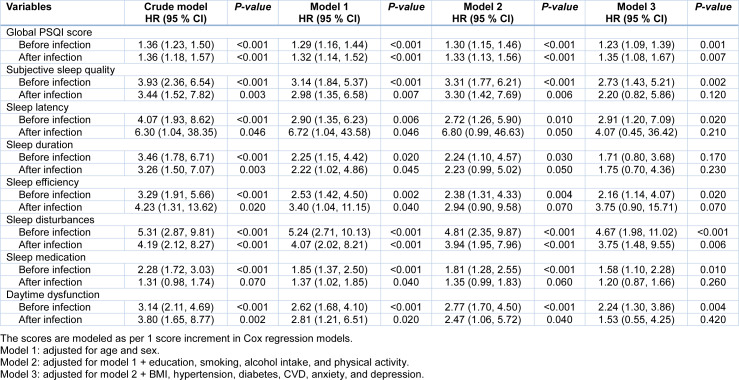
Association of the PSQI score and its seven domains with risk of progression to severe or critical pneumonia among hospitalized patients with moderate COVID-19
